# What is the benefit of associating a blockade ilioinguinal and local anesthetic infiltration in elderly patients undergoing hernia repair surgery: a double-blind randomized study

**DOI:** 10.1186/1471-2482-13-S1-A48

**Published:** 2013-09-16

**Authors:** Luana Vessicchio, Maria Luisa Mingione, Giuseppe Dimarzio, A d’Elia, Giuseppe Izzo, Francesca Grassia, Biagio Lettieri

**Affiliations:** 1 Dept. of Anesth. Surgical and Emergency Sciences-Intensive Care Unit - II University of Naples, Italy

## Introduction

Among the different anesthetic techniques used to attend hernioplasty current evidence supports the use of local anesthesia by infiltration because it determines a shorter post-operative recovery, fewer complications and lower overall costs 1. However, despite these advantages the local anesthesia by infiltration is rarely used 2. In order to improve intraoperative analgesia ilioinguinal block combined with an infiltration of local anesthetic can represent the rational, but there are no data from randomized trials to support this approach in elderly patients. The aim of our study conducted in double-blind, randomized, and is to verify if the additional ilioinguinal blockade could improve the intra-operative analgesia in the intervention of inguinal hernia repair performed under local anesthesia by infiltration.

## Materials and methods

64 patients who underwent elective surgery for inguinal hernia repair with gradual infiltration of local anesthetic were randomized in 2 groups, in double-blind. In the first group the patients were subjected to an additional ilioinguinal block with 10 ml of bupivacaine 0.25%, and others patients were subjected with isotonic saline to placebo effect. All operations of hernia repair were performed by an experienced team of surgeons. The criteria for inclusion in the study were: first intervention of inguinal hernia repair, age over 70 years, ASA class and comorbidities homogeneous. Were excluded patients with bilateral hernias, with chronic preoperative pain, dementia, and BMI greater than 29. It has not been used premedication. The intraoperative sedation with midazolam (1-2 mg) was administered when necessary. Twenty minutes before the intervention an ilioinguinal block with 10 ml of bupivacaine 0.25% was performed by injection to 3-4 cm medially from the anterior superior iliac spine in a group of patients, while the other group was treated with placebo. Immediately after surgery was assessed intra-operative pain with a visual analogue pain scale (VAS) and again 24 and 48 hours after surgery. And then was evaluated the use of analgesics (paracetamol, ketorolac and tramadol). For the post-operative pain were used paracetamol lg every 6 hours, ketorolac 30 mg every 8 hours, tramadol 50 mg, if needed.

## Results

Between the two groups there were no differences in age, use of sedation and amount of bupivacaine infiltration anesthesia. The intra-operative dose of midazolam for sedation was low (l.0 mg) and similar in both groups.

**Figure 1:**
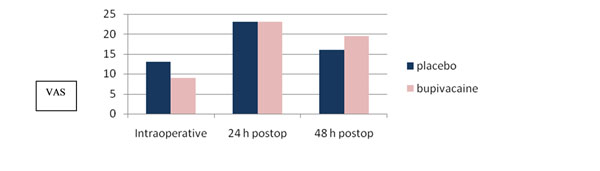


**Figure 2:**
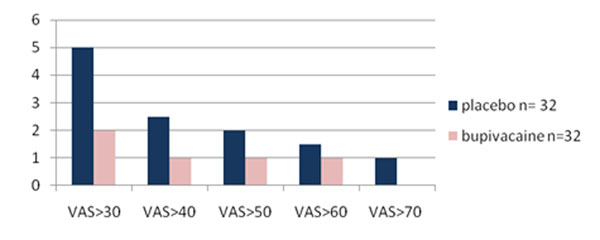


The median VAS score intra-operative was 9 in the group treated with additional ilioinguinal block while it was 13 in the group treated with placebo ilioinguinal block (P = 0.02) (Figure 1). There was no difference in pain scores or analgesic requirements at 24 and 48 hours after surgery (Figure 1). The distribution of intra-operative VAS scores showed a greater number (P <0.05) of patients with VAS score ≥ 30 intra-operative in the placebo group compared to patients treated with ilioinguinal block (Figure 2).

The results of this study have shown that the further randomized ilioinguinal block preoperative together with a gradual procedure for local infiltration anesthesia improves the intra-operative analgesia during repair of the inguinal hernia using prosthesis. These findings may have important clinical implications as it is well known that the infiltration of local anesthetic may be the most convenient technique for the repair of the inguinal hernia 3. However, this technique is not widely used, probably because of the risk of intra-operative pain, and causes of surgical preferences, and the traditional use of monitored anesthesia with propofol and opioids for short-term action 4. The results of this study demonstrate that a block ilioinguinal can provide better postoperative pain relief after inguinal hernia repair in elderly patients compared to placebo. The improved intra-operative pain associated with ilioinguinal block does not have, however, effect on the subsequent (24-48 hours) pain scores or analgesic use. In conclusion, the additional use of a preoperative ilioinguinal block the procedure of local infiltration anesthesia for inguinal hernia repair improves the intra-operative pain relief and is therefore recommended. This technique can support more widespread use of local anesthesia for inguinal hernia repair.
